# PRESERVE: adding variable flip-angle excitation to transverse relaxation-optimized NMR spectroscopy

**DOI:** 10.5194/mr-5-131-2024

**Published:** 2024-09-12

**Authors:** Bernhard Brutscher

**Affiliations:** 1 Institut de Biologie Structurale (IBS), Université Grenoble Alpes, CEA, CNRS, 71 avenue des Martyrs, 38044 Grenoble CEDEX 9, France

## Abstract

We introduce the “Polarization Restoring Excitation SEquence foR Versatile Experiments” (PRESERVE) pulse sequence element, allowing variable flip-angle adjustment in 2D ${}^{1}$ H– ${}^{15}$ N and ${}^{1}$ H– ${}^{13}$ C transverse-relaxation-optimized-spectroscopy (TROSY)-type correlation experiments. PRESERVE-TROSY exploits a remarkable array of up to nine orthogonal coherence-transfer pathways, showcasing the remarkable potential of spin manipulations achievable through the design and optimization of nuclear magnetic resonance (NMR) pulse sequences.

## 1 Introduction

**Figure 1:**
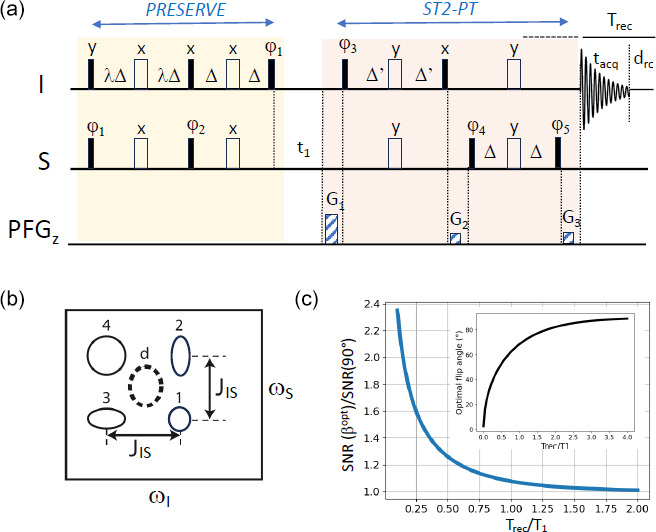
PRESERVE-TROSY experiment for recording heteronuclear 2D I – S correlation spectra. **(a)** The pulse sequence comprises the PRESERVE, $t_{1}$ , and ST2-PT building blocks. Filled and open pulse symbols correspond to 90 and 180° radio frequency (rf) pulses, respectively. The appropriate single-transition spin states are selected by adjusting the pulse phases $\phi_{3}$ and $\phi_{5}$ as well as the gradients G ${}_{1}$ , G ${}_{2}$ , and G ${}_{3}$ . Echo/anti-echo quadrature detection in $t_{1}$ is achieved by the pulsed-field gradients G ${}_{1}$ , G ${}_{2}$ , and G ${}_{3}$ with (G ${}_{1}-G_{2}$ )/(G ${}_{3}$ -G ${}_{2}$ ) $=\pm \gamma_{I}/\gamma_{S}$ as well as by inverting phases $\phi_{3}$ and $\phi_{5}$ for the anti-echo component. Phase inversion of the whole PRESERVE sequence by 180° along with the receiver phase improves the line shapes of the detected NMR signals. In addition, axial-peak suppression can be realized by inverting phases $\phi_{4}$ and $\phi_{5}$ along with the receiver phase. The heteronuclear transfer delay is set to Δ= 1/(4J ${}_{IS})$ , while Δ ' should be selected to be slightly shorter in order to reduce the intensity of residual anti-TROSY components (Schulte-Herbrüggen and Sørensen, 2000). Small flip-angle ( β ) excitation of I - and S -spin polarization is achieved by tuning the scaling factor λ to λ=β/90° . **(b)** Multiplet structure of NMR peaks detected in a scalar coupled I – S spin system, corresponding to the individual single-transition states of the I and S spins. This multiplet pattern can be reduced to a single peak using either heteronuclear spin decoupling (HSQC: peak d) or spin-state selection (TROSY: either one of the four peaks). **(c)** Numerical simulation of the expected sensitivity gain if an optimal flip angle is chosen instead of the common 90° nutation, as a function of the $T_{\mathrm{rec}}$ / $T_{1}$ ratio. The inset shows the computed optimal flip angle as a function of the $T_{\mathrm{rec}}$ / $T_{1}$ ratio according to Eq. (1). These simulations neglect spin relaxation and pulse imperfections.

Transverse relaxation-optimized spectroscopy (TROSY), pioneered by Pervushin and co-workers in 1997 (Pervushin et al., 1997), provides a powerful tool for biomolecular nuclear magnetic resonance (NMR) studies of proteins (and other macromolecules), in particular at high static magnetic field strengths. The major pulse sequence block of TROSY is the so-called ST2-PT (Pervushin et al., 1998b) or double-S ${}^{3}$ CT (Sørensen et al., 1997) sequence (Fig. 1a). Designed for heteronuclear scalar-coupled two-spin systems ( I – S ), this sequence effectively transforms single-transition states of spin S ( $S^{+}I^{\alpha }$ or $S^{+}I^{\beta }$ ) into single-transition states of spin I ( $I^{+}S^{\alpha }$ or $I^{+}S^{\beta }$ ). The relaxation properties of the single-transition states are influenced by cross-correlation between the chemical shift anisotropy (CSA) of the transverse (active) spin and the dipolar (DD) interaction between the two spins (Brutscher, 2000; Goldman, 1984). For nuclear spins with a sizeable CSA, selection of the narrowest multiplet component by the ST2-PT sequence therefore provides an increased spectral resolution. Moreover, under favourable conditions, it also offers improved experimental sensitivity compared with heteronuclear single-quantum coherence (HSQC) variants, where the passive spin is decoupled during chemical shift evolution, $t_{1}$ (Fig. 1b). The extent of line narrowing achieved via CSA–DD cross-correlation is dependent on the magnetic field, with maximal efficiency when the two spin interactions exhibit identical strengths (taking into account the projection of the CSA interaction on the I – S vector). However, spin-state selection inherent to TROSY results in a factor of 2 signal loss relative to decoupled HSQC variants. This loss needs to be compensated for by the line narrowing induced by the CSA–DD cross-correlation to yield a net sensitivity gain.

The ST2-PT sequence (and thus the TROSY experiment) has some particularly interesting features. First, it allows for sensitivity-enhanced quadrature detection; this means that both of the orthogonal S -spin components $S_{x}$ and $S_{y}$ (and *2S* ${}_{x}I_{z}$ and *2S* ${}_{y}I_{z}$ ), present after a free evolution delay $t_{1}$ , are transferred into detectable antiphase (or in-phase) coherence *2I* ${}_{x}S_{z}$ and *2I* ${}_{y}S_{z}$ ( $I_{x}$ and $I_{y}$ ) in a so-called echo/anti-echo detection mode (Kay et al., 1994; Palmer et al., 1992; Weigelt, 1998). Second, as the single-transition operators, $S_{x}I^{\alpha }=\left(S_{x}+2S_{x}I_{z}\right)/2$ and $S_{x}I^{\beta }=\left(S_{x}-2S_{x}I_{z}\right)/2$ are linear combinations of the Cartesian spin operators $S_{x}$ and *2S* ${}_{x}I_{z}$ , the equilibrium polarization of the two spin reservoirs ( I and S ) can be exploited via two parallel coherence-transfer pathways, in which the spin polarization of S ( $S_{z}$ ) is transferred into $S_{x}$ in-phase coherence, while spin polarization of I ( $I_{z}$ ) is transferred into *2S* ${}_{x}I_{z}$ antiphase coherence, or vice versa, using, for example, an insensitive nuclei enhanced by polarization transfer (INEPT)-type sequence (Brutscher et al., 1998; Pervushin et al., 1998a). Finally, I -spin polarization ( $I_{z})$ that builds up during $t_{1}$ via longitudinal spin-relaxation processes is converted by the ST2-PT sequence into S -spin polarization ( $S_{z}$ ) that contributes to the detected NMR signal during the subsequent scan (pulse sequence repetition) (Favier and Brutscher, 2011). This last feature is particularly interesting in the context of band-selective-excitation short-transient transverse relaxation-optimized spectroscopy (BEST-TROSY) experiments (Farjon et al., 2009; Favier and Brutscher, 2011; Solyom et al., 2013) where short inter-scan delays are employed, thereby minimizing relaxation-induced losses of the “over-polarized” (higher than thermally polarized) S spins. Therefore, in total, five complementary orthogonal coherence transfer pathways contribute to the detected signal in TROSY- and BEST-TROSY-type experiments.

In order to make TROSY experiments even more versatile, it would be appealing to implement the option of variable flip-angle excitation to further increase the steady-state spin polarization at high repetition rates. The concept of using non-90° nutation angles ( β ) as a means to maximize the sensitivity in a single-pulse experiment goes back to Richard Ernst and the early days of Fourier transform NMR (Ernst and Anderson, 1966). The nutation angle $\beta^{\mathrm{opt}}$ that provides the highest sensitivity is usually called the Ernst angle, and it can be shown that, for the simple case of an NMR experiment consisting of a single excitation radio frequency (rf) pulse followed by signal detection and a recovery period (recycle delay), it fulfils the following relation:

1 $$\mathrm{cos}\beta^{\mathrm{opt}}\;=\exp\left(-T_{\mathrm{rec}}/T_{1}\right),$$

where $T_{\mathrm{rec}}$ is the spin polarization recovery time (composed of the signal acquisition time, $t_{\mathrm{acq}}$ , and the recycle delay $d_{\mathrm{rc}}$ ) and $T_{1}$ is the effective longitudinal relaxation time of the excited spins (Fig. 1c). Transferring this concept to multi-pulse sequences employing a series of 90 and 180° pulses is a non-trivial task. So far, it has been adapted to heteronuclear multiple-quantum coherence (HMQC)- and heteronuclear multiple-bond correlation (HMBC)-type heteronuclear correlation experiments that require a single I -spin excitation pulse followed by one or several 180° pulses. In this case, Ernst-angle excitation can still be realized by adjusting the nutation flip angle of the initial excitation pulse to $a^{\mathrm{opt}}=\beta^{\mathrm{opt}}+n\times 180{}^{\circ}$ , where n is the number of additional 180° pulses. This has allowed the development of band-selective, optimized flip-angle short-transient (SOFAST)-HMQC and its variants (Gal et al., 2007; Kern et al., 2008; Koos and Luy, 2019; Mueller, 2008; Schanda et al., 2005; Schanda and Brutscher, 2005), for fast and sensitive recording of 2D ${}^{1}$ H– ${}^{15}$ N and ${}^{1}$ H– ${}^{13}$ C correlation spectra, as well as no relaxation delay (NORD) NMR spectroscopy (Nagy et al., 2021).

## 2 PRESERVE-TROSY

Implementing Ernst-angle excitation in TROSY-type experiments is particularly challenging, because, ideally, it requires a pulse sequence that preserves part of the steady-state polarization for both the I - and S -spin reservoirs. Nonetheless, we present a pulse scheme that fulfils this task. In the pulse sequence depicted in Fig. 1a, the initial INEPT transfer scheme of a TROSY experiment has been replaced by an alternative building block that we will refer to as “Polarization Restoring Excitation SEquence foR Versatile Experiments” (PRESERVE). It allows the adjustment of the effective excitation flip angle β for both the I - and S -spin polarizations by setting the scaling factor of the initial transfer delays to λ=β/90° . Of course, only a single effective excitation angle β can be chosen for both the I and S spins, which, in case of large differences in the longitudinal relaxation efficiency, means that it cannot be adjusted to its theoretical optimum value for both spin species simultaneously. The sin β part of I and S polarization is then transferred, as usual, to single-quantum S -spin coherence, while the remaining cos $\beta I_{z}$ and cos $\beta S_{z}$ parts are “preserved” as spin polarization or two-spin order, as will be explained in more detail below. The main coherence transfer pathways of a TROSY experiment that contribute to the detected NMR signal are conserved in this PRESERVE-TROSY experiment, with the only difference being that I -spin polarization ( $I_{z}$ ) is transferred into in-phase coherence ( $\pm S_{x}$ ), while S -spin polarization ( $S_{z})$ is transferred into antiphase coherence ( $\pm 2S_{x}I_{z}$ ) by the PRESERVE pulse sequence element. For proper functioning of this sequence, all pulse phases need to be adjusted carefully in order to ensure that the appropriate coherences (single-transition states) are selected and that all coherence transfer pathways add constructively to the recorded NMR signal. In the following, we will discuss how this can be achieved for ${}^{1}$ H– ${}^{15}$ N and ${}^{1}$ H– ${}^{13}$ C spin systems.

### 2.1 ${}^{1}$ H– ${}^{15}$ N PRESERVE-TROSY

**Figure 2:**
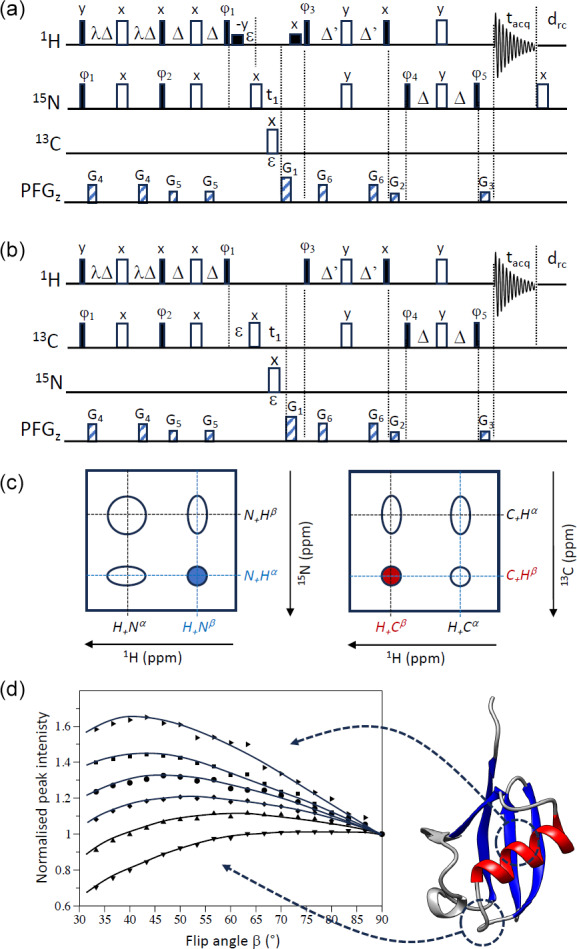
PRESERVE-TROSY pulse sequences for **(a)** ${}^{1}$ H– ${}^{15}$ N and **(b)** ${}^{1}$ H– ${}^{13}$ C spin systems. The two pulse sequences differ in the additional 180° pulse after signal detection that is only required for sequence **(a)** and the initial phase setting for $\phi_{5}$ . In addition, selective ${}^{1}$ H 90° pulses (small black squares) are added in the ${}^{1}$ H– ${}^{15}$ N version **(a)** for water flip-back (WFB) purposes (Grzesiek and Bax, 1993). The initial phase settings are as follows: **(a)** $\phi_{1}=x$ , $\phi_{2}=x$ , $\phi_{3}=y$ , $\phi_{4}=x$ , and $\phi_{5}=y$ and **(b)** $\phi_{5}=-y$ . Phase cycling and quadrature detection is performed as explained in Fig. 1. **(c)** Assignment of individual doublet lines in ${}^{1}$ H– ${}^{15}$ N and ${}^{1}$ H– ${}^{13}$ C spectra to the corresponding single-transition spin operators (in accordance with our pulse sequence simulations). The peak selected by sequences **(a)** and **(b)** is highlighted in blue ( ${}^{1}$ H– ${}^{15}$ N) and red ( ${}^{1}$ H– ${}^{13}$ C), respectively. **(d)** Peak intensities of representative amide groups, extracted from ${}^{1}$ H– ${}^{15}$ N WFB-PRESERVE-TROSY spectra recorded for uniformly ${}^{2}$ H/ ${}^{13}$ C/ ${}^{15}$ N-labelled ubiquitin at 25 °C (950 MHz) with $d_{\mathrm{rc}}=0$ ( $T_{\mathrm{rec}}=t_{\mathrm{acq}}=70$ ms) plotted as a function of the excitation flip angle β . All intensity curves are normalized to 1 for β=90° . A cartoon structure of ubiquitin is shown on the right side of panel **(d)**.

For ${}^{1}$ H– ${}^{15}$ N spin systems, the lower right cross peak (Fig. 2c) benefits from line narrowing induced by CSA–DD cross-correlation in both the $\omega_{N}$ and $\omega_{H}$ spectral dimensions. Selection of this multiplet component, often called the TROSY peak, imposes particular phase settings on the ST2-PT pulse sequence block and the coherence selection gradients (Fig. 2a). While there is general consensus on the TROSY peak to be selected and the required phase settings, there are discrepancies in the literature concerning the assignment of this TROSY peak to the corresponding single-transition spin states. This may be due to different definitions of the signs of frequencies and phases as well as their translation into actual spin rotations in product operator space (Levitt, 1997; Levitt and Johannessen, 2000). Throughout this paper, we will consider that the spectrometer phases are independent of the gyromagnetic ratio ( γ ) of a given spin species; thus, the same pulse phase induces rotations of opposite sense for nuclear spins with positive or negative γ .

The ${}^{1}$ H– ${}^{15}$ N version of PRESERVE-TROSY is depicted in Fig. 2a. The pulse sequence includes additional water flip-back pulses to make it applicable to samples in aqueous solution, which will not be further discussed in this section. The ${}^{1}$ H– ${}^{15}$ N spin system is characterized by gyromagnetic ratios of opposite sign ( $\gamma_{H}>0$ and $\gamma_{N}<0$ ) and a negative scalar coupling ( $J_{\mathrm{NH}}<0$ ). The ${}^{1}$ H- and ${}^{15}$ N-spin polarizations are split by the PRESERVE sequence into separate pathways with amplitudes cos β and sin β , respectively. The sin β parts of PRESERVE are the “active” pathways that are detected at the end of the pulse sequence and give rise to the TROSY peak:

2 $$\begin{array}{rl} H_{z} & \;N_{z}\overset{\text{PRESERVE}(\mathrm{sin}\beta )}{⟶}-H^{\alpha }N_{x}\overset{\pi N_{x}}{⟶}-H^{\alpha }N_{x}\\ & \overset{ST2-PT}{⟶}+H_{x}N^{\beta }\;\left(\frac{M_{H}+M_{N}}{2}\mathrm{sin}\beta \right). \end{array}$$

The normalized amplitude (or transfer efficiency) of these two pathways is given in parenthesis at the end, where $M_{H}$ and $M_{N}$ correspond to the steady-state magnetization of ${}^{1}$ H and ${}^{15}$ N, respectively, and spin-relaxation effects have been neglected. For simplicity, we do not consider spin evolution during $t_{1}$ here, resulting in similar pathways for the y components of the single-transition spin operators (Zuiderweg and Rousaki, 2011).

In the following, we will concentrate on the additional coherence-transfer pathways that are enabled by PRESERVE if λ<1 is chosen for the initial transfer step:

3 $$\begin{array}{rl} H_{z} & \overset{\text{PRESERVE}(\mathrm{cos}\beta )}{⟶}\;-2H_{z}N_{z}\overset{\pi N_{x}}{⟶}+2H_{z}N_{z}\\ & \overset{ST2-PT}{⟶}+H_{z}\;\;\left(M_{H}\mathrm{cos}\beta \right)\;. \end{array}$$

The unused ${}^{1}$ H-spin polarization is, thus, stored as $H_{z}$ during the inter-scan delay, similar to a small-flip-angle single-pulse experiment. The situation becomes a little more complex for the ${}^{15}$ N-spin polarization that undergoes the following transformations:

4 $$\begin{array}{rl} N_{z} & \overset{\text{PRESERVE}(\mathrm{cos}\beta )}{⟶}-N_{z}\overset{\pi N_{x}}{⟶}+N_{z}\overset{ST2-PT}{⟶}-2H_{z}N_{z}\\ & \overset{\pi N_{x}}{⟶}+2H_{z}N_{z}\left(M_{N}\mathrm{cos}\beta \right). \end{array}$$

The unused ${}^{15}$ N-spin polarization is converted into two-spin order which does not directly contribute to the NMR signal in the subsequent scan but rather requires a second reshuffling by the pulse sequence during the next scan:

5 $$\begin{array}{rl} +2H_{z} & N_{z}\left(M_{N}\mathrm{cos}\beta \right)\overset{\text{PRESERVE}}{⟶}+H_{z}\overset{\pi N_{x}}{⟶}+H_{z}\\ & \overset{ST2-PT}{⟶}-N_{z}\overset{\pi N_{x}}{⟶}+N_{z}\;\left(M_{N}\mathrm{cos}\beta \right). \end{array}$$

The cos β part of the ${}^{15}$ N-spin polarization needs to pass the PRESERVE-TROSY sequence twice before again becoming ${}^{15}$ N-spin polarization that contributes to the NMR signal of the following scan. Note that an additional 180° ${}^{15}$ N pulse is required at the end of the signal detection period in order to make this pulse sequence function properly.

Furthermore, as already described previously (Favier and Brutscher, 2011), ${}^{1}$ H polarization that builds up during $t_{1}$ due to longitudinal ${}^{1}$ H-spin relaxation will also be converted into ${}^{15}$ N polarization by the ST2-PT sequence, thereby contributing to the “active” coherence transfer pathway in the subsequent scan:

6 $$\ldots \overset{{}^{1}H\text{-}\mathrm{spin}\;\mathrm{relaxation}\;(t1)}{⟶}\;\;\;+H_{z}\overset{ST2-PT}{⟶}-N_{z}\overset{\pi N_{x}}{⟶}+N_{z}.$$

In a very similar way, ${}^{15}$ N polarization created during $t_{1}$ by spin-lattice relaxation is not lost; rather, it will be reinjected as two-spin order in the following scan:

7 $$\begin{array}{rl} \ldots & \overset{{}^{15}N\text{-}\mathrm{spin}\;\;\mathrm{relaxation}\;(t1)}{⟶}\;\;\;+N_{z}\overset{ST2-PT}{⟶}-2H_{z}N_{z}\\ & \overset{\pi N_{x}}{⟶}+2H_{z}N_{z}. \end{array}$$

In practice, although conceptually interesting, this last pathway will not contribute significantly to the detected NMR signal because of the low gyromagnetic ratio $\gamma_{N}$ ( $\gamma_{H}\approx 10\gamma_{N}$ ) and the long longitudinal relaxation times of ${}^{15}$ N, especially at high magnetic field strengths.

For echo/anti-echo quadrature detection in $t_{1}$ , the $\phi_{1}$ phases of the PRESERVE sequence have to be inverted in concert with phases $\phi_{3}$ and $\phi_{5}$ of the ST2-PT pulse sequence part. These synchronized phase adjustments preserve the outcomes of the supplementary coherence transfer pathways introduced in Eqs. (3)–(5).

### 2.2 ${}^{1}$ H– ${}^{13}$ C PRESERVE-TROSY

For ${}^{1}$ H– ${}^{13}$ C spin systems that are characterized by positive gyromagnetic ratios ( $\gamma_{H}>0$ and $\gamma_{C}>0$ ) and a scalar coupling of positive sign ( $J_{\mathrm{CH}}>0$ ), slightly different phase settings are required. Line narrowing (broadening) of ${}^{1}$ H– ${}^{13}$ C moieties in proteins and nucleic acids can be substantial in the ${}^{13}$ C dimension, while little difference in line width is observed in the ${}^{1}$ H dimension at currently available high magnetic field strengths. The line narrowing effect is particularly important for aromatic ${}^{1}$ H– ${}^{13}$ C moieties in protein side chains or nucleic acid bases. Therefore, either one of the ${}^{1}$ H single-transition spin states ( $H_{x}C^{\alpha }$ or $H_{x}C^{\beta }$ ) may be selected by a TROSY-type experiment (Brutscher et al., 1998). A ${}^{1}$ H– ${}^{13}$ C version of PRESERVE-TROSY is shown in Fig. 2b that selects the lower left component of the C–H multiplet (Fig. 2c). The coherence-transfer pathways relevant for the ${}^{1}$ H– ${}^{13}$ C PRESERVE-TROSY experiment are as follows:

8Hz,Cz⟶PRESERVE(sin⁡β)+HβCx⟶πCx+HβCx⟶ST2-PT+HxCβMH+MC2sin⁡β,9Hz⟶PRESERVE(cos⁡β)+2HzCz⟶πCx-2HzCz⟶ST2-PT+HzMHcos⁡β,10Cz⟶PRESERVE(cos⁡β)-Cz⟶πCx+Cz⟶ST2-PT-2HzCzMCcos⁡β,11-2HzCzMCcos⁡β⟶PRESERVE+Hz⟶πCx+Hz⟶ST2-PT+CzMCcos⁡β,12…⟶1H-spinrelaxation(t1)+Hz⟶ST2-PT+Cz,13…⟶13C-spinrelaxation(t1)+Cz⟶ST2-PT-2HzCz.

Again, all coherence-transfer pathways add up in a coherent way, contributing to the detected NMR signal either directly or after passing the sequence several times. Contrary to the ${}^{1}$ H– ${}^{15}$ N version, no additional 180° ${}^{13}$ C pulse is required after the NMR signal detection period.

## 3 PRESERVE: Ernst angle and experimental sensitivity

The experimental sensitivity of an experiment, defined as the NMR signal recorded in a fixed amount of time (neglecting spin relaxation) is given by the following:

14 $$\mathrm{SNR}\left(T_{\mathrm{rec}}/T_{1},\;\beta \right)=\frac{\left(1-\mathrm{exp}\left(-T_{\mathrm{rec}}/T_{1}\right)\mathrm{sin}\beta \right)}{1-\mathrm{cos}\beta \mathrm{exp}\left(-T_{\mathrm{rec}}/T_{1}\right)\sqrt{T_{\mathrm{rec}}+T_{\mathrm{Seq}}}},$$

where $T_{\mathrm{rec}}=t_{\mathrm{acq}}+d_{\mathrm{rc}}$ is the effective recycle delay, during which spin-lattice relaxation occurs; $T_{1}$ is the longitudinal relaxation time; and $T_{\mathrm{seq}}$ the length of the pulse sequence that does not contribute to the spin polarization build-up. As already indicated above, this function reaches its maximum if $\mathrm{cos}\beta^{\mathrm{opt}}=\exp\left(-T_{\mathrm{rec}}/T_{1}\right)$ (Eq. 1). Therefore, a 90° excitation angle is only close to optimal if a recycle delay $T_{\mathrm{rec}}>4T_{1}$ is chosen (Fig. 1c). In all other cases, Eq. (14) predicts a gain in experimental sensitivity if an excitation angle β<90° is chosen. The sensitivity gain achieved with Ernst-angle excitation compared with standard 90° pulse excitation becomes significant in the context of fast-pulsing NMR experiments using recycle delays that are much shorter than the longitudinal relaxation times of the excited spins (Fig. 1c). For example, if the recycle delay is 4 times shorter than the longitudinal relaxation time, the expected sensitivity gain amounts to about 60 %.

In case of the PRESERVE-TROSY experiment, this theoretical sensitivity gain becomes reduced by spin-relaxation-induced signal losses during the different pulse sequence elements (PRESERVE, $t_{1}$ , and ST2-PT) and the recycle delay as well as by other experimental imperfections, such as B ${}_{1}$ -field inhomogeneities. Therefore, the exact outcome will depend on the particular application (e.g. sample, magnetic field strength, and spin system). In general, the experiment is expected to be most attractive for I – S spin systems characterized by long longitudinal relaxation times for both I and S and for applications aiming at short overall experimental times (short recycle delays).

In order to illustrate this “new” feature of optimizing experimental sensitivity in TROSY experiments at low $T_{\mathrm{rec}}/T_{1}$ ratios using the PRESERVE sequence element, we have recorded a series of 2D ${}^{1}$ H– ${}^{15}$ N WFB-PRESERVE-TROSY spectra on a triple-labelled ( ${}^{13}$ C/ ${}^{15}$ N/ ${}^{2}$ H) sample of ubiquitin at a high magnetic field strength (950 MHz) and without additional recycle delay ( $d_{\mathrm{rc}}=0$ ). The deuteration of the protein ensures long ${}^{1}$ H longitudinal relaxation times for amide protons protected from solvent exchange. Characteristic examples of measured peak intensities for individual amide sites of ubiquitin as a function of the effective excitation flip angle β are shown in Fig. 2d. The maxima of these intensity curves are detected at flip angles ranging from about 40 to 90°. This reflects the spread in the longitudinal ${}^{1}$ H (and eventually the ${}^{15}$ N) relaxation times ( $T_{1}$ ) in our ubiquitin sample, with the shortest $T_{1}$ values observed for amide groups in the disordered parts (optimal flip angle close to 90°) and the longest ones in the well-structured parts of the protein (optimal flip angle ≪90° ). A sensitivity gain of more than 60 % is achieved for the amide sites experiencing the slowest ${}^{1}$ H longitudinal relaxation. Overall, the sensitivity gains that we observe experimentally for optimal flip-angle adjustment are in good agreement with theory (Fig. 1c), highlighting the performance of PRESERVE-TROSY to restore “unused” ${}^{1}$ H and ${}^{15}$ N polarization for subsequent scans.

**Figure 3:**
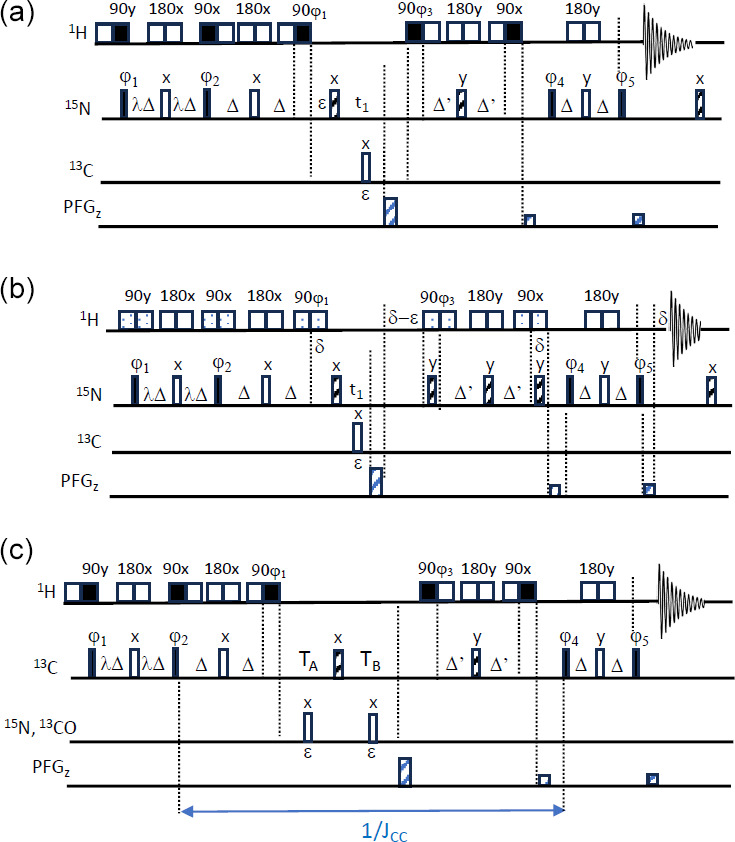
SOFAST-TROSY pulse sequences. **(a, b)** ${}^{1}$ H– ${}^{15}$ N SOFAST-TROSY. Band-selective ${}^{1}$ H pulses are applied with E-BURP-2 (90°) (Geen and Freeman, 1991) and RE-BURP (180°) (Geen and Freeman, 1991) (sequence A) or PC9 (90°) (Kupce and Freeman, 1993) and RE-BURP (180°) shapes (sequence B), while broadband ${}^{15}$ N pulses are performed using broadband UREV2 (Manu et al., 2023) (open bars) and BIP (Smith et al., 2001) (dashed bars) pulse shapes. The transfer delays Δ are adjusted to $\Delta =1/(4J_{\mathrm{NH}}$ ) (with Δ ' being slightly shorter). The initial phase settings are as follows: $\phi_{1}=x$ , $\phi_{2}=x$ , $\phi_{3}=y$ , $\phi_{4}=x$ , and $\phi_{5}=y$ . Phase cycling and quadrature detection is performed as described in Fig. 1. **(c)** ${}^{1}$ H– ${}^{13}$ C SOFAST-TROSY. Band-selective 90 and 180° ${}^{1}$ H pulses are applied with E-BURP2 and RE-BURP profiles. For conventional ${}^{13}$ C editing, $T_{A}=\varepsilon$ and $T_{B}=t_{1}+\varepsilon$ , while for CT editing, $T_{A}=1/(2J_{CC})-2\Delta -t_{1}/2$ and $T_{B}=1/(2J_{CC})-2\Delta$ ' $+t_{1}/2$ . The initial phase settings are as follows: $\phi_{1}=x$ , $\phi_{2}=x$ , $\phi_{3}=y$ , $\phi_{4}=x$ , and $\phi_{5}=-y$ . Phase cycling and quadrature detection is performed as described in Fig. 1.

## 4 SOFAST-TROSY: combining longitudinal relaxation enhancement with variable flip-angle excitation

An alternative approach to enhance the experimental sensitivity in TROSY NMR experiments and to allow for high repetition rates (short inter-scan delays) is the use of longitudinal relaxation enhancement (LRE) techniques (Brutscher and Solyom, 2017; Pervushin et al., 2002; Schanda, 2009). The most prominent and widely used example is arguably the BEST-TROSY implementation. In band-selective-excitation short-transient (BEST) experiments (Schanda et al., 2006), the high-power, squared ${}^{1}$ H pulse shapes are replaced by amplitude- and/or phase-modulated pulse shapes that allow for the excitation of only a subset of ${}^{1}$ H spins that are spectrally well separated, while leaving all others (outside this spectral region) close to equilibrium. During the inter-scan delay, the ${}^{1}$ H spins in thermal equilibrium then act as a low-energy reservoir that can “absorb” energy from the excited (high-energy) ${}^{1}$ H via either dipolar relaxation or hydrogen exchange mechanisms.

Although LRE will reduce the interest in variable flip-angle excitation, we implemented the PRESERVE building block into a BEST-TROSY experiment that we will refer to as SOFAST-TROSY. Figure 3 shows different implementations of SOFAST-TROSY, optimized for ${}^{1}$ H– ${}^{15}$ N (Fig. 3a, b) or ${}^{1}$ H– ${}^{13}$ C (Fig. 3c) spin systems. The ${}^{1}$ H– ${}^{15}$ N SOFAST-TROSY sequences differ with respect to the choice of the band-selective ${}^{1}$ H pulse shapes. Figure 3a uses E-BURP2 (Geen and Freeman, 1991) pulse shapes for ${}^{1}$ H 90° excitation and flip-back pulses, resulting in a more compact (shorter) pulse sequence, while symmetric (universal rotation) 90° pulse shapes (PC9) (Kupce and Freeman, 1993) were preferred in the pulse sequence of Fig. 3b due to their cleaner excitation profile. In the case of short inter-scan delays, even small perturbations of the “passive” ${}^{1}$ H spins can have a significant impact on longitudinal ${}^{1}$ H-spin relaxation (Schanda, 2009). The pulse shapes are represented by their binary replacement schemes (Lescop et al., 2010): open squares account for chemical shift and J -coupling evolution during the pulse duration, while filled squares indicate the time at which the effective spin rotation of either 90 or 180° takes place. For ${}^{1}$ H– ${}^{13}$ C SOFAST-TROSY, E-BURP-2 pulse shapes were chosen for the ${}^{1}$ H 90° pulses, in order to allow correct tuning of the heteronuclear transfer delays in the presence of (relatively) long band-selective pulses of typically a few milliseconds and $J_{\mathrm{CH}}$ coupling constants ranging from 125 to 200 Hz. In addition, the sequence in Fig. 3c includes the option of CT ${}^{13}$ C frequency editing in $t_{1}$ , which is of interest for ${}^{1}$ H– ${}^{13}$ C spin systems in uniformly ${}^{13}$ C-labelled proteins or nucleic acids.

**Figure 4:**
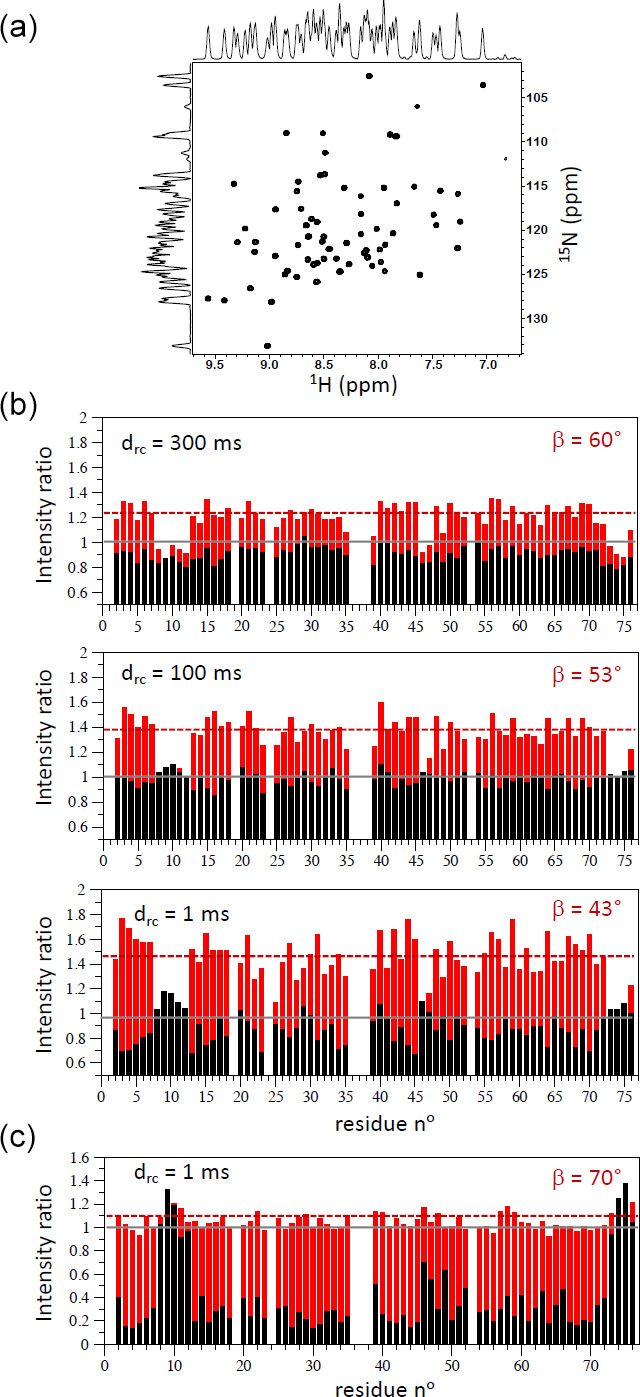
${}^{1}$ H– ${}^{15}$ N SOFAST-TROSY data of ubiquitin. **(a)** ${}^{1}$ H– ${}^{15}$ N spectrum recorded at 600 MHz on a uniformly ${}^{13}$ C/ ${}^{15}$ N-labelled sample of ubiquitin at 25 °C with $t_{\mathrm{acq}}=70$ ms, $d_{\mathrm{rc}}=1$ ms, and β=70° . **(b)** Intensity ratios measured for a perdeuterated and ${}^{13}$ C/ ${}^{15}$ N-labelled sample of ubiquitin at 25 °C (950 MHz) with SOFAST-TROSY/BEST-TROSY (red bars) and WFB-TROSY/BEST-TROSY (black bars). The results obtained for different inter-scan delays and experimentally optimized flip angles are shown. Panel **(c)** shows similar data but recorded on a fully protonated ${}^{13}$ C/ ${}^{15}$ N-labelled sample of ubiquitin and a single inter-scan delay $d_{\mathrm{rc}}=1$ ms.

**Figure 5:**
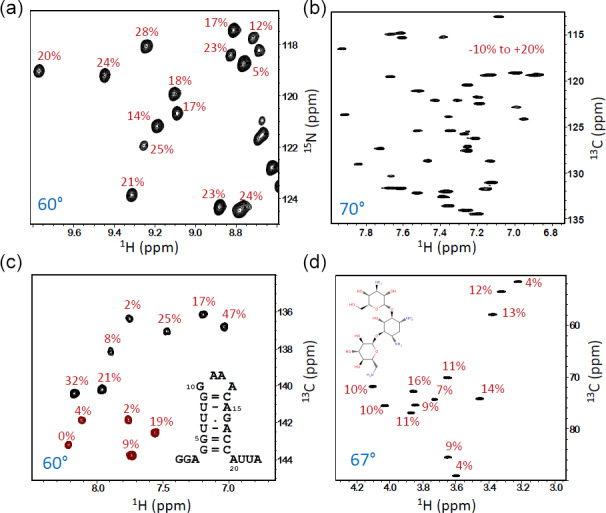
SOFAST-TROSY spectra of different samples, all recorded at 600 or 700 MHz ( ${}^{1}$ H frequency) with an inter-scan delay $d_{\mathrm{rc}}=1$ ms. **(a)** A small part of the ${}^{1}$ H– ${}^{15}$ N spectrum of ${}^{13}$ C/ ${}^{15}$ N/ ${}^{2}$ H-labelled SiR (18 kDa, deuteration level ∼60% , 30 °C, and $t_{\mathrm{acq}}=70$ ms). **(b)** Aromatic ${}^{1}$ H– ${}^{13}$ C lysozyme spectrum at natural ${}^{13}$ C abundance and high sample concentration (2 mM) recorded at 40 °C ( $t_{\mathrm{acq}}=100$ ms). **(c)** ${}^{1}$ H– ${}^{13}$ C CT spectrum of a 23-nucleotide RNA hairpin with uniformly ${}^{13}$ C/ ${}^{15}$ N-labelled guanine (G) and uracil (U) nucleotides (25 °C, $t_{\mathrm{acq}}=100$ ms). Positive peaks (black contours) are from G, while negative peaks (red) originate from U nucleotides. **(d)** ${}^{1}$ H– ${}^{13}$ C spectrum of the antibiotic kanamycin at natural ${}^{13}$ C abundance and high sample concentration ( ∼10 mM) recorded at 30 °C ( $t_{\mathrm{acq}}=100$ ms). The numbers (in red) indicate the NMR signal ratio between data recorded with the optimized flip angle (indicated in blue) compared with a data set recorded under identical conditions but with β=90° .

First, we applied the ${}^{1}$ H– ${}^{15}$ N SOFAST-TROSY experiment to uniformly ${}^{13}$ C/ ${}^{15}$ N-labelled and ${}^{13}$ C/ ${}^{15}$ N/ ${}^{2}$ H-labelled samples of ubiquitin (25 °C). For both samples, the pulse sequence of Fig. 3b was preferred, due to the slightly higher sensitivity at short recycle delays. Figure 4a illustrates the spectral quality (line shapes) obtained. The performance in terms of experimental sensitivity can be appreciated from the graphs in Fig. 4b ( ${}^{13}$ C/ ${}^{15}$ N/ ${}^{2}$ H ubiquitin) and Fig. 4c ( ${}^{13}$ C/ ${}^{15}$ N ubiquitin). We recorded ${}^{1}$ H– ${}^{15}$ N SOFAST-TROSY spectra for different recycle delays ( $d_{\mathrm{rc}}$ ) and flip angles ( β ) optimized for the structured parts of the protein and then compared them to conventional BEST-TROSY (Favier and Brutscher, 2011) and WFB-TROSY (Loria et al., 1999) pulse sequences. The BEST-TROSY data were taken as a reference, and the intensity ratios of SOFAST-TROSY and WFB-TROSY with respect to this reference are plotted in Fig. 4 using red and black bars, respectively. In the case of the perdeuterated ubiquitin sample, BEST-TROSY and WFB-TROSY perform about equally well, and average sensitivity gains of 25 %–50 %, depending on the chosen inter-scan delay, are observed for SOFAST-TROSY. In the case of a fully protonated ubiquitin sample, the situation is quite different. The intensity of individual correlation peaks increases by up to 8-fold when replacing a WFB-TROSY sequence with a BEST-TROSY sequence at very short recycle delays, while only a minor further improvement ( ∼10% ) is achieved by SOFAST-TROSY. This observation can be rationalized by the fact that LRE reduces the effective longitudinal ${}^{1}$ H relaxation in BEST-TROSY and SOFAST-TROSY to less than 200 ms, bringing $T_{1}$ close to $T_{\mathrm{rec}}$ . Thus, the observed sensitivity increase of <15% (depending on the specific amide site) is again in good agreement with the theoretical expectation for $T_{\mathrm{rec}}/T_{1}\approx 1$ (Fig. 1c).

Figure 5 shows some further applications of the SOFAST-TROSY experiment to molecular systems with different sizes and natures, with all spectra recorded without any additional recycle delay. The first example is SiR (Sibille et al., 2005), a protein of 18 kDa that is uniformly enriched in ${}^{13}$ C and ${}^{15}$ N as well as partially deuterated ( ∼60% ). The ${}^{1}$ H– ${}^{15}$ N spectra were recorded with the pulse sequence of Fig. 3b, and a flip angle β≈60° was found to provide the highest average experimental sensitivity. Compared with an experiment recorded with a 90° excitation flip angle, a sensitivity increase of ∼20% was observed (Fig. 5a). The remaining applications concern ${}^{1}$ H– ${}^{13}$ C correlation spectra of the aromatic side chains in lysozyme, a 14 kDa protein at natural ${}^{13}$ C abundance (Fig. 5b); a small RNA hairpin with uniform ${}^{13}$ C and ${}^{15}$ N labelling of U and G nucleotides (Fig. 5c) (Rennella et al., 2017); and, finally, the antibiotic kanamycin, an aminoglycoside composed of three sugar units (Fig. 5d). For these ${}^{1}$ H– ${}^{13}$ C correlation experiments, the sequence of Fig. 3c was employed with standard (non-CT) ${}^{13}$ C editing for the two natural abundance samples, whereas CT ${}^{13}$ C editing was used for the uniformly ${}^{13}$ C-labelled RNA hairpin. In all three cases, a nominal flip excitation angle of 60–70° was found to be optimal in terms of (average) experimental sensitivity. The sensitivity gain with respect to a β≈90° reference spectrum, indicative of the longitudinal relaxation times of the ${}^{1}$ H and ${}^{13}$ C ( ${}^{15}$ N) spins, is different for each of the investigated molecular systems with significant variations observed from one nuclear site to the other.

## 5 Conclusions

In this work, we have introduced the PRESERVE pulse sequence element, which allows variable flip-angle adjustment in 2D ${}^{1}$ H– ${}^{15}$ N and ${}^{1}$ H– ${}^{13}$ C TROSY-type correlation experiments. Compatible with both hard-pulse-based TROSY versions and shaped-pulse BEST-type implementations, PRESERVE enables versatile applications. PRESERVE-TROSY and SOFAST-TROSY experiments stand out among the wealth of available solution NMR experiments by exploiting a remarkable array of up to nine parallel coherence-transfer pathways in Cartesian-spin-operator space. Thus, these experiments exemplify the remarkable flexibility of nuclear spin manipulations achievable through the design and optimization of NMR pulse sequences. Our focus has been on the theoretical elucidation and experimental realization of the PRESERVE concept. By employing a minimal two-step phase cycle, clean ${}^{1}$ H– ${}^{15}$ N and ${}^{1}$ H– ${}^{13}$ C correlation spectra can be acquired in short experimental time, making these experiments, in principle, suitable for a broad range of molecular systems possessing H–N- or H–C spin systems. Beyond their conceptual elegance, PRESERVE-TROSY and SOFAST-TROSY provide valuable additions to the NMR toolbox. Whether PRESERVE-TROSY and SOFAST-TROSY experiments yield sensitivity enhancements under fast-pulsing conditions is contingent upon various factors, such as the molecular size, isotope labelling scheme, and magnetic field strength, determining the relaxation properties of the sample under investigation. As a rule of thumb, these experiments will be particularly beneficial for molecular systems characterized by prolonged spin-lattice relaxation times of both correlated nuclear spins. Extension of the PRESERVE-TROSY concept to higher-dimensional triple-resonance correlation experiments is possible and currently under development in our laboratory.

## Supplement

10.5194/mr-5-131-2024-supplement The supplement related to this article is available online at: https://doi.org/10.5194/mr-5-131-2024-supplement.

## Supplement

10.5194/mr-5-131-2024-supplement
10.5194/mr-5-131-2024-supplement The supplement related to this article is available online at: https://doi.org/10.5194/mr-5-131-2024-supplement.

## Data Availability

The presented pulse sequences are provided in the Supplement, and they will also be included in the NMRlib package that is freely available at https://www.ibs.fr/en/communication-outreach/scientific-output/software/nmrlib-2-0-ibs-pulse-sequence-tools-for-bruker-spectrometers (last access: 11 September 2024). NMR data/spectra can be accessed at 10.5281/zenodo.11447367 (Brutscher, 2024).

## Appendix

The supplement related to this article is available online at: https://doi.org/10.5194/mr-5-131-2024-supplement.
