# Supplementary material for: PRESERVE: adding variable flip-angle excitation to transverse relaxation-optimized NMR spectroscopy
Source: Magn Reson (Gott). 2024 Sep 12;5(2):131–42. doi: 10.5194/mr-5-131-2024 (PMC12178132; doi:10.5194/mr-5-131-2024)
Supplement: The supplement related to this article is available online at: https://doi.org/10.5194/mr-5-131-2024-supplement. [file mr-5-131-2024-supplement.pdf]

Supplement of Magn. Reson., 5, 131–142, 2024
https://doi.org/10.5194/mr-5-131-2024-supplement
© Author(s) 2024. CC BY 4.0 License.

Supplement of

PRESERVE: adding variable flip-angle excitation to transverse relaxation-optimized NMR spectroscopy

Bernhard Brutscher

Correspondence to: Bernhard Brutscher (bernhard.brutscher@ibs.fr)

The copyright of individual parts of the supplement might differ from the article licence.

1. Bruker pulse sequence code: HN water-flip-back PRESERVE-TROSY

```
;HN_WFB_PRESERVE_TROSY
;BB 2024
;
;
;$CLASS=IBS
;$DIM=2D
;$TYPE=
;$SUBTYPE=
;$COMMENT=


prosol relations=<IBS>


#include <Avance.incl>
#include <Grad.incl>
#include <Delay.incl>



define list<gradient> EA3 = { 1.0000 0.8750 }
define list<gradient> EA5 = { 0.6667 1.0000 }
define list<gradient> EA7 = { 1.0000 0.6595 }

/*******************************************************/
/*  Predefined shapes for 1H pulses       *************/
/*******************************************************/

/*  water pulse  (p48, pl48)   */
"p48=1m"
"plw48=plw1*(pow((p1/p48),2))"

"p16=1000u"
"p17=300u"

/
*******************************************************************/
/*   13C Adiabatic pulse
*/
/
*******************************************************************/

"p8=200u"
"spoff13=0.0" /*  BIP offset */
"spw13=plw2*(pow((p3*8/p8),2))"   /* BIP power level  */

/
*******************************************************************/
/*   calculation of shaped 15N pulse parameters
*/
/
*******************************************************************/
"p50 =500u"    /* BIP pulse length  */
"spoff50=0.0" /*  BIP offset */
"spw50=plw3*(pow((p21*8/p50),2))"   /* BIP power level  */

"p51=4.875/(40*bf3/1000000)" /* REBURP pulse length  */
"spw51=plw3*(pow((p21*1.97/p51)/0.0798,2))"   /* REBURP power level
*/
"spoff51=0.0"                /* REBURP offset */
"spoal51=0.5"

"p52 =p21*22"    /* UREV2 pulse length  */
"spoff52=0.0" /*  UREV2 offset */
"spw52=plw3"   /* UREV2 power level  */
"spoal52=0.5"

/
*******************************************************************/
/*   DELAYS
*/
/
*******************************************************************/

"d11=30m"

;"d25=2.6m"
"d26=2.7m"
"d27=2.5m"  /* set slightly shorter than d26 for relaxation
compensation  */

"DELTA1=d25-p21-p17-d16"
"DELTA2=d27-p17-d16-p21"
"DELTA3=d26-p17-d16-p21"
"DELTA4=d26-p21-p17-d16"


#   ifdef LABEL_CN
"DELTA=p8+d0*2"
#   else
"DELTA=d0*2"
#   endif /*LABEL_CN*/


/
*******************************************************************/
/*   time increments in 15N dimension                              */
/
*******************************************************************/
"d0=0"
"in0=inf1/2"

"acqt0=0"
baseopt_echo


1 d11 ze
2 d11
```

```
3 5u pl1:f1 pl2:f2 pl3:f3
  (p50:sp50 ph8):f3
  d1  pl3:f3
  50u UNBLKGRAD
/***************************************/
/*   H-N transfer (PRESERVE)          */
/***************************************/
 (ralign (p1 ph10) (p21 ph13):f3 )
  p17:gp1
  d16
  DELTA1
  (center (p1*2 ph1) (p21*2 ph1):f3 )
  DELTA1 pl3:f3
  p17:gp1
  d16

  (lalign (p1 ph11) (p21 ph14):f3 )

  DELTA4
  p17:gp2
  d16

  (center (p1*2 ph1) (p21*2 ph1):f3 )
  p17:gp2
  d16
  DELTA4

  (p1 ph12)

  3u pl48:f1
  (p48 ph15)
  3u pl1:f1

/***************************************/
/*   15N labeling                      */
/***************************************/
  d0

#   ifdef LABEL_CN
  (p8:sp13 ph1):f2
#   else
#   endif /*LABEL_CN*/

  d0
  (p50:sp50 ph1):f3
  DELTA

  p16:gp3*EA3
  d16

/***************************************/
/*   TROSY-type H-N back transfer     */
/***************************************/
  3u pl48:f1
```

```
  (p48 ph1)
  3u pl1:f1
  (p1 ph6)
  p17:gp4
  d16
  DELTA2 pl1:f1
  (center (p1*2 ph2) (p50:sp50 ph2):f3 )
  DELTA2  pl3:f3
  p17:gp4
  d16
  3u pl1:f1
  (p1 ph1)
/***************************************/

  p16:gp5*EA5
  d16

/***************************************/
  (p21 ph9):f3
  p17:gp6
  d16
  DELTA3
  (center (p1*2 ph2) (p51:sp51 ph2):f3 )
  DELTA3 pl3:f3
  p17:gp6
  d16
  (p21 ph7):f3
/***************************************/

  p16:gp7*EA7
  d16  BLKGRAD
/***************************************/
/*   Signal detection & looping       */
/***************************************/
  go=2 ph31
  d11 mc #0 to 2
     F1EA(calgrad(EA3) & calgrad(EA5) & calgrad(EA7) & calph(ph6,
+180) & calph(ph7, +180) & calph(ph12, +180) & calph(ph13, +180),
caldel(d0, +in0) & calph(ph7, +180) & calph(ph9, +180) & calph(ph31,
+180))
exit

ph1=0
ph2=3
ph3=2
ph4=3
ph5=0
ph6=1
ph7=1 1 3 3
ph8=0
ph9=0 0 2 2
ph10=1 3
ph11=0 2
ph12=0 2
ph13=0 2
ph14=0 2
ph15=3 1
ph31=0 2 2 0
```

2 Bruker pulse sequence code: HN SOFAST-TROSY (PC9, REBURP)

```
;HN_SOFAST_TROSY_PC9_UREV2
;BB 2024
;
;
;$CLASS=IBS
;$DIM=2D
;$TYPE=
;$SUBTYPE=
;$COMMENT=


prosol relations=<IBS>


#include <Avance.incl>
#include <Grad.incl>
#include <Delay.incl>



define list<gradient> EA3 = { 1.0000 0.8750 }
define list<gradient> EA5 = { 0.6667 1.0000 }
define list<gradient> EA7 = { 1.0000 0.6595 }

/*******************************************************/
/*  Predefined shapes for 1H pulses       *************/
/*  cnst1: center of excitation band     **************/
/*  cnst2: excitation band width         **************/
/*******************************************************/

/*  PC9 (p41, sp25)   */
"p41=7.2/(cnst2*bf1/1000000)" /*  PC9  pulse length  */
"spw25=plw1*(pow((p1*1.01/p41)/0.125,2))" /* PC9  power level  */
"spoff25=bf1*(cnst1/1000000)-o1"  /*  PC9  offset */
"spoal25=0.5"

/*  REBURP (p42, sp26)   */

"p42=4.875/(cnst2*bf1/1000000)" /* REBURP pulse length  */
"spw26=plw1*(pow((p1*1.97/p42)/0.0798,2))"   /* REBURP power level
*/
"spoff26=bf1*(cnst1/1000000)-o1" /* REBURP offset */
"spoal26=0.5"

/*  EBURP & EBURP_TR  (p43, sp28, sp29)   */

"p43=4.6/(cnst2*bf1/1000000)" /*  EBURP pulse length   */
"spw28=plw1*(pow((p1*1.04/p43)/0.06103,2))"   /* EBURP power level
*/
"spw29=plw1*(pow((p1*1.04/p43)/0.06103,2))"   /* EBURP power level
*/
"spoff28=bf1*(cnst1/1000000)-o1" /*  EBURP offset */
"spoal28=0"
"spoff29=bf1*(cnst1/1000000)-o1" /*  EBURP_REV offset */
"spoal29=1.0"

"p16=1000u"
"p17=300u"

/
*******************************************************************/
/*   13C Adiabatic pulse
*/
/
*******************************************************************/

"p8=200u"
"spoff13=0.0" /*  BIP offset */
"spw13=plw2*(pow((p3*8/p8),2))"   /* BIP power level  */

/
*******************************************************************/
/*   calculation of shaped 15N pulse parameters
*/
/
*******************************************************************/
"p50 =500u"    /* BIP pulse length  */
"spoff50=0.0" /*  BIP offset */
"spw50=plw3*(pow((p21*8/p50),2))"   /* BIP power level  */

"p51=4.875/(50*bf3/1000000)" /* REBURP pulse length  */
"spw51=plw3*(pow((p21*1.97/p51)/0.0798,2))"   /* REBURP power level
*/
"spoff51=0.0"                 /* REBURP offset */
"spoal51=0.5"

"p52 =p21*22"    /* UREV2 pulse length  */
"spoff52=0.0" /*  UREV2 offset */
"spw52=plw3"   /* UREV2 power level  */
"spoal52=0.5"


/
*******************************************************************/
/*   DELAYS
*/
/
*******************************************************************/

"d11=30m"

;"d25=2.6m"
"d26=2.7m"
"d27=2.5m"  /* set slightly shorter than d26 for relaxation
compensation  */
"d28=(p41*0.5-p51)*0.5"
"d29=(p41*1.5-p51)*0.5"
```

```
"DELTA1=d25-p41*0.5-p42*0.5"
"DELTA2=d27-p17-d16-p42*0.5-p41*0.5"
"DELTA3=d26-p17-d16-p42*0.5"
"DELTA4=d26-p41*0.5-p42*0.5"
"DELTA5=p41*0.51"

/
*******************************************************************/
/*   time increments in 15N dimension                              */
/
*******************************************************************/
"d0=3u"
"in0=inf1/2"

#   ifdef LABEL_CN
"DELTA=p41*0.51-(d0*2+p8)"
#   else
"DELTA=p41*0.51-(d0*2)"
#   endif /*LABEL_CN*/



"acqt0=0"
baseopt_echo


1 d11 ze
2 d11
3 5u pl1:f1 pl2:f2 pl3:f3
  (p50:sp50 ph8):f3
  d1 pl3:f3
  50u UNBLKGRAD
/***************************************/
/*   H-N transfer (PRESERVE)          */
/***************************************/

  (center (p41:sp25 ph10) (p21 ph13):f3 )  /* PC9 */

  DELTA1
  (center (p42:sp26 ph1) (p52:sp52 ph1):f3 )

  DELTA1  pl3:f3

  (center (p41:sp25 ph11) (p21 ph14):f3 )    /* PC9 */

  DELTA4
  (center (p42:sp26 ph1) (p52:sp52 ph1):f3 )
  DELTA4

  (p41:sp25 ph12)   /* PC9 */

/***************************************/
/*   15N labeling                      */
/***************************************/
  (p50:sp50 ph1):f3
  d0

#   ifdef LABEL_CN
  (p8:sp13 ph1):f2
#   else
#   endif /*LABEL_CN*/

  d0
  DELTA

  p16:gp3*EA3
  d16

/***************************************/
/*   TROSY-type H-N back transfer     */
/***************************************/
  (lalign (p41:sp25 ph6) (p50:sp50 ph2):f3 )
  p17:gp4
  d16
  DELTA2
  (center (p42:sp26 ph2) (p50:sp50 ph2):f3 )
  DELTA2  pl3:f3
  p17:gp4
  d16
  (ralign (p41:sp25 ph1) (p50:sp50 ph2):f3 )
/***************************************/

  p16:gp5*EA5
  d16 pl3:f3

/***************************************/
  (p21 ph9):f3
  p17:gp6
  d16
  DELTA3
  (center (p42:sp26 ph2) (p52:sp52 ph2):f3 )
  DELTA3 pl3:f3
  p17:gp6
  d16
  (p21 ph7):f3
/***************************************/
  DELTA5

  p16:gp7*EA7
  d16  BLKGRAD
/***************************************/
/*   Signal detection & looping       */
/***************************************/
  go=2 ph31
  d11 mc #0 to 2
     F1EA(calgrad(EA3) & calgrad(EA5) & calgrad(EA7) & calph(ph6,
+180) & calph(ph7, +180)& calph(ph12, +180) & calph(ph13, +180),
caldel(d0, +in0) & calph(ph7, +180) & calph(ph9, +180)& calph(ph31,
```

```
+180))
exit


ph1=0
ph2=1
ph4=3
ph6=3
ph7=1 1 3 3
ph8=0
ph9=0 0 2 2
ph10=1 3
ph11=0 2
ph12=0 2
ph13=0 2
ph14=0 2
ph31=0 2 2 0


;pl0 : 0W
;pl1 : f1 channel - power level for pulse (default)
;pl3 : f3 channel - power level for pulse (default)
;p16:  gradient pulse                         [1 msec]
;p17:  gradient pulse
;p21: f3 channel -  90 degree high power pulse
;d0 : incremented delay (F1)                           [3 usec]
;d1 : relaxation delay
;d11: delay for disk I/O                               [30 msec]
;d16: delay for gradient recovery
;d25: 1/(4J(NH)*lambda - for variable flip angle excitation
;d26: 1/(4J(NH)
;d27: 1/(4J(NH)
;cnst1: H(N) excitation frequency (in ppm)
;cnst2: H(N) excitation band width (in ppm)
;inf1: 1/SW(N) = 2 * DW(N)
;in0: 1/(2 * SW(N)) = DW(N)
;nd0: 2
;ns: 2 * n
;ds: >= 16
;td1: number of experiments in F1
;FnMODE: echo-antiecho in F1
```

3 Bruker pulse sequence code: HN SOFAST-TROSY (EBURP2, REBURP)

```
;HN_SOFAST_TROSY_EBURP_UREV2
;BB 2024
;
;$CLASS=IBS
;$DIM=2D
;$TYPE=
;$SUBTYPE=
;$COMMENT=


prosol relations=<IBS>


#include <Avance.incl>
#include <Grad.incl>
#include <Delay.incl>



define list<gradient> EA3 = { 1.0000 0.8750 }
define list<gradient> EA5 = { 0.6667 1.0000 }
define list<gradient> EA7 = { 1.0000 0.6595 }

/*******************************************************/
/*  Predefined shapes for 1H pulses       *************/
/*  cnst1: center of excitation band     **************/
/*  cnst2: excitation band width         **************/
/*******************************************************/

/*  PC9 (p41, sp25)   */
"p41=7.2/(cnst2*bf1/1000000)" /*  PC9  pulse length  */
"spw25=plw1*(pow((p1*1.01/p41)/0.125,2))" /* PC9  power level  */
"spoff25=bf1*(cnst1/1000000)-o1"  /*  PC9  offset */
"spoal25=0.5"

/*  REBURP (p42, sp26)   */

"p42=4.875/(cnst2*bf1/1000000)" /* REBURP pulse length  */
"spw26=plw1*(pow((p1*1.97/p42)/0.0798,2))"   /* REBURP power level
*/
"spoff26=bf1*(cnst1/1000000)-o1" /* REBURP offset */
"spoal26=0.5"

/*  EBURP2 EBURP2_TR  (p43, sp28, sp29)   */

"p43=4.6/(cnst2*bf1/1000000)"
"spw28=plw1*(pow((p1*1.04/p43)/0.06103,2))"
"spw29=plw1*(pow((p1*1.04/p43)/0.06103,2))"
"spoff28=bf1*(cnst1/1000000)-o1"
"spoal28=0"
"spoff29=bf1*(cnst1/1000000)-o1"
"spoal29=1.0"

"p16=1000u"
```

```
"p17=300u"

/
********************************************************************/
/*   13C Adiabatic pulse
*/
/
********************************************************************/

"p8=200u" /* BIP pulse length  */
"spoff13=0.0" /*  BIP offset */
"spw13=plw2*(pow((p3*8/p8),2))"   /* BIP power level  */

/
********************************************************************/
/*   calculation of shaped 15N pulse parameters
*/
/
********************************************************************/
"p50 =500u"    /* BIP pulse length  */
"spoff50=0.0" /*  BIP offset */
"spw50=plw3*(pow((p21*8/p50),2))"   /* BIP power level  */
"spoal50=0.5"

"p51=4.875/(50*bf3/1000000)" /* REBURP pulse length  */
"spw51=plw3*(pow((p21*1.97/p51)/0.0798,2))"   /* REBURP power level
*/
"spoff51=0.0"                /* REBURP offset */
"spoal51=0.5"

"p52 =p21*22"    /* UREV2 pulse length  */
"spoff52=0.0" /*  UREV2 offset */
"spw52=plw3"   /* UREV2 power level  */
"spoal52=0.5"

/
********************************************************************/
/*   DELAYS
*/
/
********************************************************************/

"d11=30m"

;"d25=2.6m"
"d28=2.6m"
"d26=2.7m"
"d27=2.5m"  /* set slightly shorter than d26 for relaxation
compensation  */

"DELTA1=d25-p42*0.5"
"DELTA4=d28-p42*0.5-p43*0.5-p17-d16"

"DELTA2=d27-p17-d16-p42*0.5-p43*0.5"
```

```
"DELTA3=d26-p17-d16-p42*0.5"


/
*********************************************************************/
/*   time incremennts in 15N dimension
*/
/
*********************************************************************/
"d0=0"
"in0=inf1/2"

#   ifdef LABEL_CN
"DELTA=d0*2+p8"
#   else
"DELTA=d0*2"
#   endif /*LABEL_CN*/


"acqt0=0"
baseopt_echo


1 d11 ze
2 d11
3 5u pl1:f1 pl2:f2 pl3:f3
  (p50:sp50 ph1):f3
  d1 pl3:f3
  50u UNBLKGRAD
/***************************************/
/*   H-N transfer (PRESERVE)          */
/***************************************/

  (ralign (p43:sp28 ph10) (p21 ph13):f3 )

  DELTA1
  (center (p42:sp26 ph1) (p52:sp52 ph1):f3 )

  DELTA1  pl3:f3

  (lalign (p43:sp29 ph11) (p21 ph14):f3 )

  p17:gp1
  d16

  DELTA4
  (center (p42:sp26 ph1) (p52:sp52 ph1):f3 )
  p17:gp1
  d16
  DELTA4

  (p43:sp28 ph12)

/***************************************/
```

```
/*   15N labeling                      */
/***************************************/
  d0

#   ifdef LABEL_CN
  (p8:sp13 ph1):f2
#   else
#   endif /*LABEL_CN*/

  d0
  (p50:sp50 ph1):f3
  DELTA

  p16:gp3*EA3
  d16

/***************************************/
/*   TROSY-type H-N back transfer     */
/***************************************/
  (p43:sp29 ph6)   /* EBURP_REV */
  p17:gp4
  d16
  DELTA2
  (center (p42:sp26 ph2) (p50:sp50 ph2):f3 )
  DELTA2  pl3:f3
  p17:gp4
  d16
  (p43:sp28 ph1)    /* EBURP  */
/***************************************/

  p16:gp5*EA5
  d16

/***************************************/
  (p21 ph8):f3
  p17:gp6
  d16
  DELTA3
  (center (p42:sp26 ph2) (p52:sp52 ph2):f3 )
  DELTA3 pl3:f3
  p17:gp6
  d16
  (p21 ph7):f3
/***************************************/

  p16:gp7*EA7
  d16  BLKGRAD
/***************************************/
/*   Signal detection & looping       */
/***************************************/
  go=2 ph31
  d11 mc #0 to 2
     F1EA(calgrad(EA3) & calgrad(EA5) & calgrad(EA7) & calph(ph6,
+180) & calph(ph7, +180)& calph(ph12, +180) & calph(ph13, +180),
```

```
caldel(d0, +in0) & calph(ph7, +180) & calph(ph8, +180)& calph(ph31,
+180) )
exit


ph1=0
ph2=1
ph4=3
ph6=1
ph7=1 1 3 3
ph8=0 0 2 2
ph10=1 3
ph11=0 2
ph12=0 2
ph13=0 2
ph14=0 2
ph31=0 2 2 0


;pl0 : 0W
;pl1 : f1 channel - power level for pulse (default)
;pl3 : f3 channel - power level for pulse (default)
;p16:  gradient pulse                         [1 msec]
;p17:  gradient pulse
;p21: f3 channel -  90 degree high power pulse
;p22: f3 channel - 180 degree high power pulse
;d0 : incremented delay (F1)                           [3 usec]
;d1 : relaxation delay
;d11: delay for disk I/O                               [30 msec]
;d16: delay for homospoil/gradient recovery
;d25: 1/(4J(NH)*lambda - for variable flip angle excitation
;d26: 1/(4J(NH)
;d27: 1/(4J(NH)
;cnst1: H(N) excitation frequency (in ppm)
;cnst2: H(N) excitation band width (in ppm)
;inf1: 1/SW(N) = 2 * DW(N)
;in0: 1/(2 * SW(N)) = DW(N)
;nd0: 2
;ns: 2 * n
;ds: >= 16
;td1: number of experiments in F1
;FnMODE: echo-antiecho in F1
```

4 Bruker pulse sequence code: HC SOFAST-TROSY

```
;HC_SOFAST_C_TROSY_noCT
;BB 2024
;
;
;$CLASS=IBS
;$DIM=2D
;$TYPE=
;$SUBTYPE=
;$COMMENT=


prosol relations=<IBS>


#include <Avance.incl>
#include <Grad.incl>
#include <Delay.incl>


define list<gradient> EA3 = { 1.0000 0.7500 }
define list<gradient> EA5 = { 0.5000 1.0000 }
define list<gradient> EA7 = { 0.9028 0.2972 }

/*****************************************************/
/*  Predefined shapes for 1H pulses       *************/
/*  cnst1: center of excitation band     **************/
/*  cnst2: excitation band width         **************/
/*****************************************************/

/*  PC9 (p41, sp25)   */
"p41=7.2/(cnst2*bf1/1000000)" /*  PC9  pulse length  */
"spw25=plw1*(pow((p1*1.01/p41)/0.125,2))" /* PC9  power level  */
"spoff25=bf1*(cnst1/1000000)-o1"  /*  PC9  offset */
"spoal25=0.5"

/*  REBURP (p42, sp26)   */

"p42=4.875/(cnst2*bf1/1000000)" /* REBURP pulse length  */
"spw26=plw1*(pow((p1*1.97/p42)/0.0798,2))"   /* REBURP power level
*/
"spoff26=bf1*(cnst1/1000000)-o1" /* REBURP offset */
"spoal26=0.5"

/*  EBURP & EBURP_TR  (p43, sp28, sp29)   */

"p43=4.6/(cnst2*bf1/1000000)" /*  EBURP pulse length   */
"spw28=plw1*(pow((p1*1.04/p43)/0.06103,2))"   /* EBURP power level
*/
"spw29=plw1*(pow((p1*1.04/p43)/0.06103,2))"   /* EBURP power level
*/
"spoff28=bf1*(cnst1/1000000)-o1" /*  EBURP offset */
"spoal28=0"
"spoff29=bf1*(cnst1/1000000)-o1" /*  EBURP_REV offset */
"spoal29=1.0"

"p16=1000u"
"p17=300u"

/
*********************************************************************/
/*   13C pulse shapes
*/
/
*********************************************************************/
"p14=4.875/(50.0*bf2/1000000)" /* REBURP pulse length  */
"spw3=plw2*(pow((p3*1.97/p14)/0.0798,2))"   /* REBURP power level
*/
"spoal3=0.5"
"spoff3=0"

/
*********************************************************************/
"p40 =p3*8"    /* BIP pulse length  */
"spoff40=0.0" /*  BIP offset */
"spw40=plw2"   /* BIP power level  */
"spoal40=0.5"

/
*********************************************************************/
/*   calculation of shaped 15N pulse parameters
*/
/
*********************************************************************/
"p50 =500u"    /* BIP pulse length  */
"spoff50=0.0" /*  BIP offset */
"spw50=plw3*(pow((p21*8/p50),2))"   /* BIP power level  */

"p51=4.875/(50*bf3/1000000)" /* REBURP pulse length  */
"spw51=plw3*(pow((p21*1.97/p51)/0.0798,2))"   /* REBURP power level
*/
"spoff51=0.0"                 /* REBURP offset */
"spoal51=0.5"

/
*********************************************************************/
/*   DELAYS
*/
/
*********************************************************************/

"d11=30m"

;"d25=1.0m" /* adjustable delay for flip angle */
"d26=1.4m"
"d27=1.2m"  /* set slightly shorter than d26 for relaxation
compensation  */
"d28=1.4m"  /* 1/(2JCH) */
```

```
"DELTA1=d25-p42*0.5"
"DELTA2=d26-p42*0.5-p43*0.5"
"DELTA3=d27-p17-d16-p42*0.5"
"DELTA4=d28-p42*0.5-p43*0.5"

"DELTA=d0*2+p3*4/3.1416"

/
*********************************************************************/
/*   time increments in 13C dimension (semi CT)
*/
/
*********************************************************************/
"d0=3u"
"in0=inf1"

"acqt0=0"
baseopt_echo


1 d11 ze
2 d11
3 5u pl1:f1 pl2:f2 pl3:f3
  d1
  50u UNBLKGRAD
/***************************************/
/*  PRESERVE  H-C transfer            */
/***************************************/
 (ralign (p43:sp28 ph10) (p3 ph13):f2 )

  DELTA1
  (center (p42:sp26 ph1) (p14:sp3 ph1):f2 )

  DELTA1  pl2:f2

  (lalign (p43:sp29 ph11) (p3 ph14):f2 )

  DELTA4

  (center (p42:sp26 ph1) (p14:sp3 ph1):f2 )

  DELTA4

  (p43:sp28 ph12)


/***************************************/
/*   13C labeling                     */
/***************************************/
 (p40:sp40 ph1):f2
  d0

  p16:gp3*EA3
  d16 pl2:f2

/***************************************/
/*   TROSY-type H-C back transfer     */
/***************************************/
  (p43:sp29 ph6)   /* EBURP_REV */
  DELTA2
  (center (p42:sp26 ph2) (p40:sp40 ph2):f2 )
  DELTA2 pl2:f2
  (p43:sp28 ph1)    /* EBURP  */
/***************************************/

  p16:gp5*EA5
  d16

/***************************************/
  (p3 ph8):f2
  p17:gp6
  d16
  DELTA3
  (center (p42:sp26 ph2) (p14:sp3 ph2):f2 )
  DELTA3 pl2:f2
  p17:gp6
  d16
  (p3 ph7):f2
/***************************************/

  p16:gp7*EA7
  d16  BLKGRAD
/***************************************/
/*   Signal detection & looping       */
/***************************************/
  go=2 ph31
  d11 mc #0 to 2
     F1EA(calgrad(EA3) & calgrad(EA5) & calgrad(EA7) & calph(ph6,
+180) & calph(ph7, +180)& calph(ph12, +180) & calph(ph13, +180),
caldel(d0, +in0) & caldel(d31, -in31) &  calph(ph7, +180) &
calph(ph8, +180)& calph(ph31, +180))
exit


ph1=0
ph2=1
ph4=3
ph6=1
ph7=3 3 1 1
ph8=0 0 2 2
ph10=1 3
ph11=0 2
ph12=0 2
ph13=0 2
ph14=0 2
ph31=0 2 2 0
```

5 Bruker pulse sequence code: HC SOFAST-TROSY (semi-CT ^{13}C editing)

```
;HC_SOFAST_C_TROSY
;BB 2024
;
;$CLASS=IBS
;$DIM=2D
;$TYPE=
;$SUBTYPE=
;$COMMENT=


prosol relations=<IBS>


#include <Avance.incl>
#include <Grad.incl>
#include <Delay.incl>


define list<gradient> EA3 = { 1.0000 0.7500 }
define list<gradient> EA5 = { 0.5000 1.0000 }
define list<gradient> EA7 = { 0.9028 0.2972 }

/*****************************************************/
/*  Predefined shapes for 1H pulses       *************/
/*  cnst1: center of excitation band     **************/
/*  cnst2: excitation band width         **************/
/*****************************************************/

/*  PC9 (p41, sp25)   */
"p41=7.2/(cnst2*bf1/1000000)" /*  PC9  pulse length  */
"spw25=plw1*(pow((p1*1.01/p41)/0.125,2))" /* PC9  power level  */
"spoff25=bf1*(cnst1/1000000)-o1"  /*  PC9  offset */
"spoal25=0.5"

/*  REBURP (p42, sp26)   */

"p42=4.875/(cnst2*bf1/1000000)" /* REBURP pulse length  */
"spw26=plw1*(pow((p1*1.97/p42)/0.0798,2))"   /* REBURP power level
*/
"spoff26=bf1*(cnst1/1000000)-o1" /* REBURP offset */
"spoal26=0.5"

/*  EBURP & EBURP_TR  (p43, sp28, sp29)   */

"p43=4.6/(cnst2*bf1/1000000)" /*  EBURP pulse length   */
"spw28=plw1*(pow((p1*1.04/p43)/0.06103,2))"   /* EBURP power level
*/
"spw29=plw1*(pow((p1*1.04/p43)/0.06103,2))"   /* EBURP power level
*/
"spoff28=bf1*(cnst1/1000000)-o1" /*  EBURP offset */
"spoal28=0"
"spoff29=bf1*(cnst1/1000000)-o1" /*  EBURP_REV offset */
"spoal29=1.0"

"p16=1000u"
"p17=300u"

/
*********************************************************************/
/*   13C pulse shapes
*/
/
*********************************************************************/
"p14=4.875/(50.0*bf2/1000000)" /* REBURP pulse length  */
"spw3=plw2*(pow((p3*1.97/p14)/0.0798,2))"   /* REBURP power level
*/
"spoal3=0.5"
"spoff3=0"

/
*********************************************************************/
"p40 =p3*8"    /* BIP pulse length  */
"spoff40=0.0" /*  BIP offset */
"spw40=plw2"   /* BIP power level  */
"spoal40=0.5"


/
*********************************************************************/
/*   calculation of shaped 15N pulse parameters
*/
/
*********************************************************************/
"p50 =500u"    /* BIP pulse length  */
"spoff50=0.0" /*  BIP offset */
"spw50=plw3*(pow((p21*8/p50),2))"   /* BIP power level  */

"p51=4.875/(50*bf3/1000000)" /* REBURP pulse length  */
"spw51=plw3*(pow((p21*1.97/p51)/0.0798,2))"   /* REBURP power level
*/
"spoff51=0.0"                 /* REBURP offset */
"spoal51=0.5"

/
*********************************************************************/
/*   DELAYS
*/
/
*********************************************************************/

"d11=30m"

;"d25=1.0m" /* adjustable delay for flip angle */
"d26=1.4m"
"d27=1.2m"  /* set slightly shorter than d26 for relaxation
compensation  */
"d28=1.4m"  /* 1/(2JCH) */
```

```
"DELTA1=d25-p42*0.5"
"DELTA2=d26-p42*0.5-p43*0.5"
"DELTA3=d27-p17-d16-p42*0.5"
"DELTA4=d28-p42*0.5-p43*0.5"

"DELTA=d0*2+p3*4/3.1416"

/
********************************************************************
/*   time increments in 13C dimension (semi CT)
*/
/
********************************************************************
"d0=3u"

"d22=1/(2*70)"
"d24=d22-d26*2-d16-p16-p3*2/3"
"d31=d24"

"d15=td1*inf1/4"   /* t1(max)/2  */
"in30=(d15-d24)/(td1/2)"
"in31=d24/(td1/2)"
"in0=in30*2+in31"


"acqt0=0"
baseopt_echo


1 d11 ze
2 d11
3 5u pl1:f1 pl2:f2 pl3:f3
  d1
  50u UNBLKGRAD
/**************************************/
/*  PRESERVE  H-C transfer            */
/**************************************/
 (ralign (p43:sp28 ph10) (p3 ph13):f2 )

  DELTA1
  (center (p42:sp26 ph1) (p14:sp3 ph1):f2 )

  DELTA1  pl2:f2

  (lalign (p43:sp29 ph11) (p3 ph14):f2 )

  DELTA4

  (center (p42:sp26 ph1) (p14:sp3 ph1):f2 )

  DELTA4

  (p43:sp28 ph12)
```

```
/**************************************/
/*   13C labeling                     */
/**************************************/
  d0
  d24
 (p40:sp40 ph1):f2
  d31     /* d24 - t1/2  */

  p16:gp3*EA3
  d16 pl2:f2

/**************************************/
/*   TROSY-type H-C back transfer     */
/**************************************/
  (p43:sp29 ph6)   /* EBURP_REV */
  DELTA2
  (center (p42:sp26 ph2) (p40:sp40 ph2):f2 )
  DELTA2 pl2:f2
  (p43:sp28 ph1)    /* EBURP  */
/**************************************/

  p16:gp5*EA5
  d16

/**************************************/
  (p3 ph8):f2
  p17:gp6
  d16
  DELTA3
  (center (p42:sp26 ph2) (p14:sp3 ph2):f2 )
  DELTA3 pl2:f2
  p17:gp6
  d16
  (p3 ph7):f2
/**************************************/

  p16:gp7*EA7
  d16  BLKGRAD
/**************************************/
/*   Signal detection & looping       */
/**************************************/
  go=2 ph31
  d11 mc #0 to 2
     F1EA(calgrad(EA3) & calgrad(EA5) & calgrad(EA7) & calph(ph6,
+180) & calph(ph7, +180)& calph(ph12, +180) & calph(ph13, +180),
caldel(d0, +in0) & caldel(d31, -in31) &  calph(ph7, +180) &
calph(ph8, +180)& calph(ph31, +180))
exit


ph1=0
ph2=1
ph4=3
ph6=1
```

```
ph7=3 3 1 1
ph8=0 0 2 2
ph10=1 3
ph11=0 2
ph12=0 2
ph13=0 2
ph14=0 2
ph31=0 2 2 0


;pl0 : 0W
;pl1 : f1 channel - power level for pulse (default)
;pl3 : f3 channel - power level for pulse (default)
;sp13: f2 channel - shaped pulse 180 degree (Ca and C=O, adiabatic)
;p16:  gradient pulse                         [1 msec]
;p17:  gradient pulse
;p21: f3 channel -  90 degree high power pulse
;p22: f3 channel - 180 degree high power pulse
;p8 : f2 channel - 180 degree shaped pulse for inversion (adiabatic)
;d0 : incremented delay (F1)                           [3 usec]
;d1 : relaxation delay; 1-5 * T1
;d11: delay for disk I/O                               [30 msec]
;d16: delay for homospoil/gradient recovery
;d25: 1/(4J(NH)
;d26: 1/(4J(NH)
;d27: 1/(4J(NH)
;cnst1: H(N) excitation frequency (in ppm)
;cnst2: H(N) excitation band width (in ppm)
;inf1: 1/SW(N) = 2 * DW(N)
;in0: 1/(2 * SW(N)) = DW(N)
;nd0: 2
;ns: 2 * n
;ds: >= 16
;td1: number of experiments in F1
;FnMODE: echo-antiecho in F1
```
